# Terahertz Metasurface for Spatial Detection of Full Stokes Parameters and Polarization Imaging

**DOI:** 10.1002/nap2.70109

**Published:** 2026-04-27

**Authors:** Shengnan Guan, Jierong Cheng, Haifeng Xu, Fan Li, Chenglong Liu, Shengjiang Chang

**Affiliations:** ^1^ Institute of Modern Optics Nankai University Tianjin China; ^2^ Tianjin Key Laboratory of Micro‐Scale Optical Information Science and Technology Tianjin China; ^3^ Tianjin Key Laboratory of Optoelectronic Sensor and Sensing Network Technology Tianjin China

**Keywords:** metalens, polarization imaging, polarization multiplexing, spatial polarimetry, terahertz

## Abstract

Polarimetry can unveil rich multidimensional information of the optical field. Conventional approaches encounter significant challenges when analyzing complex spatially varying vectorial optical fields. In this paper, we propose an efficient polarimetry technique based on subwavelength‐scale metalenses. This technique facilitates the spatial detection of full Stokes parameters, thereby overcoming the conventional constraints imposed on the uniformity of spatial intensity and polarization. The proposed metalens achieves 4 × 4 pixels with each pixel offering 6‐channel polarization focusing, from which the polarization distribution can be calculated with good accuracy. This method provides a novel and robust technological scheme for detecting complex full Stokes parameters at the terahertz band.

## Graphical abstract

This work presents a THz polarization analyzer with significantly improved robustness against nonuniformity. By integrating four polarization‐sensitive meta‐atoms into a meta‐cell, the metalens splits incident light into six polarization components and generates off‐axis focal spots. By observing the intensity of each component, the Stokes parameters can be accurately obtained.

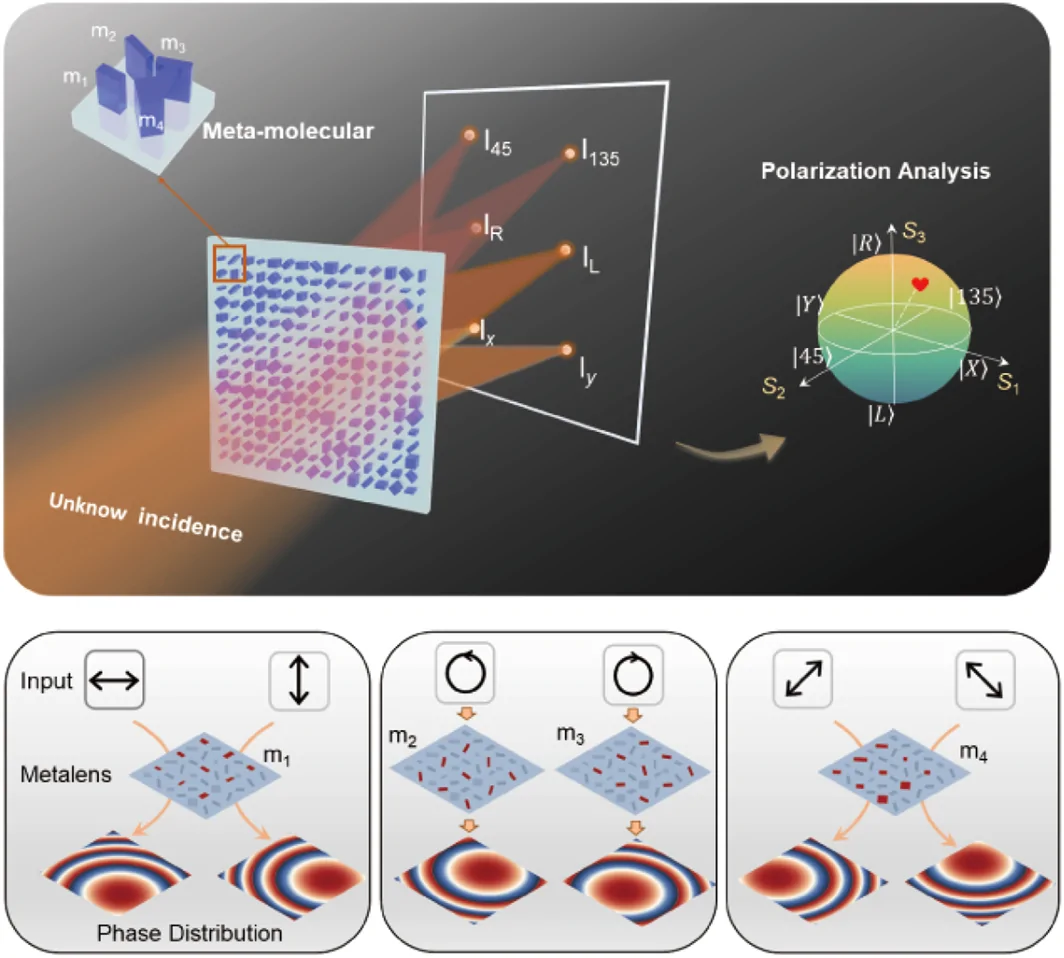

## 1 Introduction

Terahertz (THz) waves, situated between the microwave and infrared regions of the electromagnetic spectrum, exhibit significant potential in applications such as biological detection [1, 2] and 6G wireless communication [3]. The polarization state, which is traditionally defined by the orientation of the vectorial electric field, constitutes a fundamental physical property of the THz wave [4, 5, 6]. It introduces a degree of freedom in the study of light–matter interactions and thereby facilitates significant progress in analyzing crucial material properties, including birefringence, morphology, and molecular configuration. Polarization detection has demonstrated significant promise in biochemical imaging [7] and trace sensing [8]. There is a lack of mature polarization analysis instruments in the THz band. The THz detectors are either polarization‐insensitive (such as bolometers and pyroelectric detectors) or responsive to a specific polarization state (such as the photoconductive antennas). They are often unable to capture polarization information in an intuitive manner. In most cases, the acquisition of the full Stokes parameters requires the rotation of the polarizer or the detector multiple times [9]. This leads to cumbersome steps, system instability, and polarization errors. Consequently, the development of efficient methods for polarization analysis and potentially for polarization imaging in the THz band represents an urgent need.

In recent years, the emergence of metasurfaces has paved the way for polarization detection [10, 11] and polarization imaging [12, 13, 14, 15]. Metasurfaces are capable of precisely controlling the phase, amplitude, and polarization of electromagnetic waves by artificial structures. This flexibility enables applications such as metalenses [16, 17, 18], holography [19, 20], and coding metasurfaces [21, 22] with versatile vectorial field manipulating capabilities. By designing anisotropic meta‐cells, metasurfaces can separate the orthogonal linearly polarized components into two focal spots. By measuring the amplitude and phase of the two focal points, the polarization state of the incident light can be reconstructed [23]. Furthermore, some researchers have achieved polarization state measurement using pure intensity detection. For example, by performing mode purity analysis of the transmitted vortex fields and conducting multidimensional analysis of the transmitted field, complete polarization parameters of the incident field can be reconstructed [24]. Zhao et al. employed a tri‐layer metallic THz metasurface to realize an azimuthally polarized THz vector beam generator, which was further capable of polarization analysis for linearly polarized light [12]. Moreover, some pioneering research has achieved polarization state measurement in four or six polarization‐dependent focal points of an assembled large metalens, where each sub‐metalens responds to a specific polarization state and produces either a single focal spot or a pair of orthogonally polarized focal spots. Then, polarization detection is achieved by analyzing the intensity relation of all focal spots. For instance, Wei et al. developed a THz assembled metalens by rectangular‐patterned sub‐metalenses that generate four focal points, enabling polarization detection of THz beams [25]. Zhang et al. proposed a crosstalk‐free full Stokes polarimeter in the infrared regime by integrating six polarization‐related sub‐metalenses [26]. Zheng et al. proposed a rectangular‐tessellated six‐focal‐point metalens that converts all polarization states to *x*‐polarization while focusing [27]. By only measuring the intensity of the transmitted *x*‐polarized light field, complete polarization information can be acquired. In addition to rectangular tiling configurations, there are various methods for constructing assembled metalens, for instance, in a fan‐shaped [28, 29] or linear pattern [30]. These technologies offer a straightforward way of polarization characterization, yet it is limited to cases where the incoming beam has uniform polarization and uniform intensity.

In the cases that the incoming beam has a nonuniform intensity distribution or nonuniform polarization distribution, such as a vortex beam or a cylindrical vector beam, the relative intensity of the focal spots will be altered. This is because each focal spot is produced by a specific area on the metasurface. Consequently, the spatial nonuniformity gives rise to significant errors in polarization characterization [26, 27, 28, 29, 30]. In addition, the footprint of the metalens is usually large after the sub‐metalens assembly, which not only increases the system complexity but also limits the spatial resolution. Polarization‐multiplexed metalenses have proven effective for improving the robustness of polarization detection in the optical band [31]. In the terahertz regime, however, their application is hindered by an intrinsically low pixel count—typically three to four orders of magnitude lower than that of optical devices.

In this study, we propose a THz polarization analyzer with compact size and significantly improved robustness against nonuniformity. The metalens splits and focuses six polarization states, namely 0°, 90°, 45°, 135°, right‐circular polarization (RCP), and left‐circular polarization (LCP) to six focal spots. Instead of assembling sub‐metalenses, it is achieved through six‐channel polarization multiplexing in the sub‐wavelength scale metasurface units. Each unit consists of four spatially staggered anisotropic meta‐atoms that are sensitive to different polarization states. The spatial variation of the incoming beam contributes to all of the focal spots, and the relative intensity can be maintained to calculate the full Stokes parameters, allowing for precise reconstruction of the polarization state even with nonuniform beam intensity. We further construct a 4 × 4 metalens array for detecting nonuniform polarization distributions. Experimental results confirm that the metalens and the array can precisely analyze the polarization state and its distribution, respectively, through a single‐shot measurement. In addition, we extend this method to build a polarization imaging device and successfully capture six independent image displays that are sensitive to polarization. These results hold promising application prospects in the fields of THz security inspection and biochemical detection.

## 2 Principle of Polarimetry and Metasurface Design

The Stokes parameters(*S*) are widely adopted to characterize the polarization states, which can be derived by directly acquiring the intensity decomposed into six fundamental polarization states: [11]

(1) $$S=\left[\begin{array}{l} S_{0}\\ S_{1}\\ S_{2}\\ S_{3} \end{array}\right]=\left[\begin{array}{l} I_{x}+I_{y}\\ I_{x}-I_{y}\\ I_{45}+I_{135}\\ I_{R}+I_{L} \end{array}\right],$$

where *S*_0_–*S*_3_ represent four components of Stokes parameters. {*x*‐, *y*‐, 45°‐, 135°‐, *L*‐, *R*‐} are defined as six fundamental polarization states with *I*_*x*_, *I*_*y*_, *I*_45_, *I*_135_, *I*_*L*_, and *I*_*R*_ denoting the corresponding intensities, respectively. Polarization angle *α* and ellipticity *χ* can be mathematically calculated by the Stokes parameters:

(2) $$\begin{array}{l} \alpha =0.5\;\arctan\frac{S_{2}}{S_{1}}\\ \chi =0.5\;\arcsin\frac{S_{3}}{S_{0}} \end{array}.$$

The proposed metasurface polarization analyzer is shown in Figure 1a. The metasurface precisely splits the incident light into six fundamental polarization components and focuses them onto distinct off‐axis locations within the focal plane. By detecting the intensity distribution on the focal plane in a single shot, the full Stokes parameters can be obtained according to Equation (1). To achieve polarization‐dependent focusing, the design principle of the metasurface is illustrated in Figure 1b–d. A meta‐cell consists of four rectangular rods (*m*_1_∼*m*_4_) that respond to different polarization states. Among them, *m*_1_ and *m*_2_ are used to handle a pair of orthogonal linearly polarization states (*x*‐/*y*‐, 45°‐/135°‐). *m*_3_ and *m*_4_ interact with *L*‐ and *R*‐ through the geometric phase, respectively. To achieve different off‐axis foci, the four sets of rods sequentially provide the corresponding focusing phase profiles, as shown in Figure 1b–d. The phase profiles *φ*_*i*_ (*i* = 1–6 represent six fundamental polarization states) are defined by the following equation:

(3) $$\varphi_{i}\left(x,y\right)=-\frac{2\pi }{\lambda }\left(\sqrt{{\left(m\Lambda -x_{i}\right)}^{2}+{\left(n\Lambda -y_{i}\right)}^{2}+f^{2}}-f\right),$$

where *λ* is the working wavelength, *f* is the distance of the focal plane to the metalens, namely focal length, *Λ* is the period of meta‐cell, (*m*, *n*) is the meta‐cell coordinate, and (*x*_*i*_, *y*_*i*_) corresponds to the position of the focal spot in the focal plane. In this study, (*x*_*i*_, *y*_*i*_) of six polarization‐sensitive foci are sequentially located at (−1 cm, −1 cm), (1 cm, −1 cm), (−1 cm, 1 cm), (1 cm, 1 cm), (−1 cm, 0 cm), and (1 cm, 0 cm). The focal length is fixed as 5 cm, and the metasurface size is 5 × 5 cm^2^. Based on Equation (3), we determine the phase profiles for each polarization state, denoted as *φ*_*x*_, *φ*_*y*_, *φ*_45_, *φ*_135_, *φ*_*L*_, and *φ*_*R*_.

**FIGURE 1:**
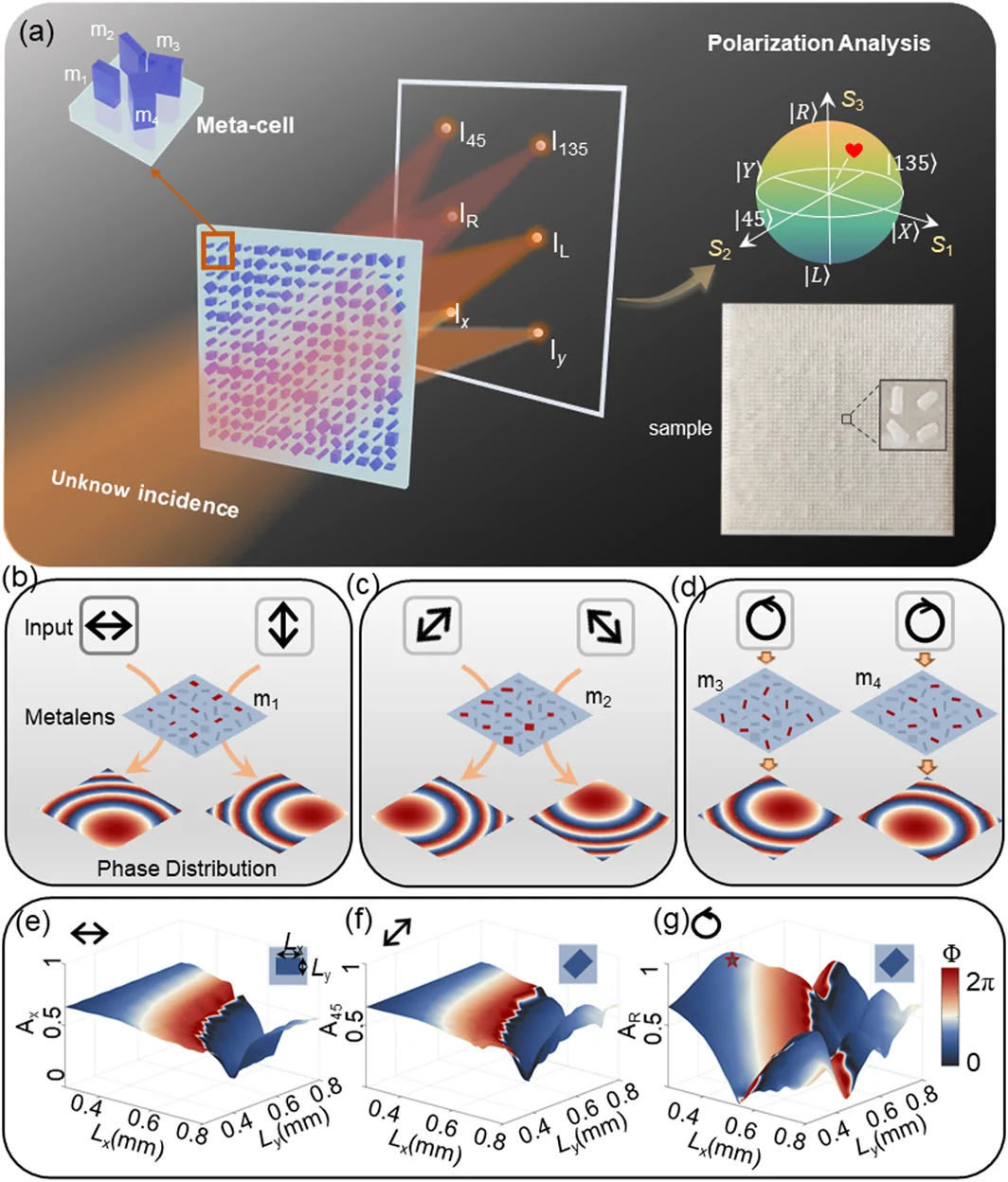
(a) The schematic diagram illustrating the polarization analyzer and the meta‐cell construction. The rods *m*_1_–*m*_4_ generate independent phase responses corresponding to different polarization states. (b) *m*_1_ is engineered for *x*‐/*y*‐ polarized light; (c) *m*_2_ is designed for 45°‐ and 135°‐polarized light with desired phase patterns in each polarization state; (d) *m*_3_ and *m*_4_ are tailored for *L*‐ and *R*‐ light. Simulated phase and amplitude response of (e) *x*‐ polarization, (f) 45°‐ polarization, (g) *R*‐ polarization by sweeping geometric parameter*s L*_*x*_ and *L*_*y*_.

In this work, the metasurface is made of alumina, with the refractive index of 3 + 0.02*i* at the working frequency of 0.14 THz. The unit period *p* = 1 mm. The heights of the rod and the substrate are both 2 mm. The commercial software FDTD is applied to simulate the transmission responses of all the rods. For *m*_1_, under *x*‐polarization excitation, the phase response and amplitude response are obtained by sweeping the length *L*_*x*_ and width *L*_*y*_ of the rectangular rod within the range of 0.2–0.8 mm. As shown in Figure 1e, the phase covers 0 to 2π. Due to the diagonal symmetry between *y*‐polarization and *x*‐polarization, the *y*‐polarization database can be obtained by transposing the *x*‐polarization database. By rotating the rectangular rod by 45° and illuminating it with 45°‐polarized light, we successfully obtained the database for 45° in Figure 1f and the transposed database for 135°, which is used to build the *m*_2_ rods. Regarding circular polarization states, Figure 1g illustrates the dependence of both the orthogonal circular polarization conversion efficiency and the phase of cross‐converted components on the dimensions of the rod. The peak efficiency is denoted by a star sign. Therefore, the optimal dimensions of *L*_*x*_ *=* 0.24 mm and *L*_*y*_ *=* 0.66 mm are chosen for *m*_3_ and *m*_4_. The orientation of *m*_3_ and *m*_4_ is determined by half of the geometric phase *φ*_*L*_/2 and *φ*_*R*_/2, respectively. The database in Figure 1e–g helps to build the metalens in a straightforward way.

## 3 Theoretical and Experimental Polarization Analysis

We first numerically simulate the field distribution in the focal plane of the metalens under six fundamental polarization excitations, as shown in Figure 2a, indicating the energy allocation on different foci. Theoretically, the energy distribution ratio among the foci follows a 1/3:0:1/6:1/6:1/6:1/6, where the excitation polarization state holds 1/3 energy, the orthogonal polarization state holds 0, and other polarization states hold 1/6, respectively. In the experiment, the designed metalens is fabricated using digital light processing (DLP) 3D printing technology. Figure 1a shows a photograph of the fabricated sample, with detailed features highlighted in the inset. Then, we modulate the polarization state of the incident light by employing a half‐wave plate and a quarter‐wave plate and measure the intensity distributions in six cases. The measured raw focal patterns are shown in Figure 2b. Slight deviations of energy distribution are observed in the experimental results. This is attributed to fabrication imperfections and inter‐element cross‐talk in the metasurface, which lead to variations in focusing efficiency among the focal points. To minimize the errors, we correct the intensity distribution by assigning a correction coefficient *γ* = [*γ*_*x*_, *γ*_*y*_, *γ*_45_, *γ*_135_, *γ*_*L*_, *γ*_*R*_].

(4) $$\gamma_{m}=I_{\text{source}}/i_{m},$$

where *I*_source_ is the source intensity and *i*_*m*_ (*m* represents the polarization state) is the *m*th focus spot intensity under *m*‐excitation polarization state, as shown in Figure 2b. The calculated correction coefficient *γ* is [1.62, 1, 1.05, 1.32, 1.1, 1.02]. It is consistent among different replicated samples and is applicable to all kinds of illuminations. The experimentally measured focal plane field distributions after correction are shown in Figure 2c, which is consistent with the theoretical results. Subsequently, to verify the effectiveness of the designed metasurface and the correction coefficient *γ*, the focal spot intensities are utilized to reconstruct Stokes parameters, and the results are marked on a Poincaré sphere in Figure 2d. The reconstructed Stokes parameters accurately reproduce the six fundamental polarization states.

**FIGURE 2:**
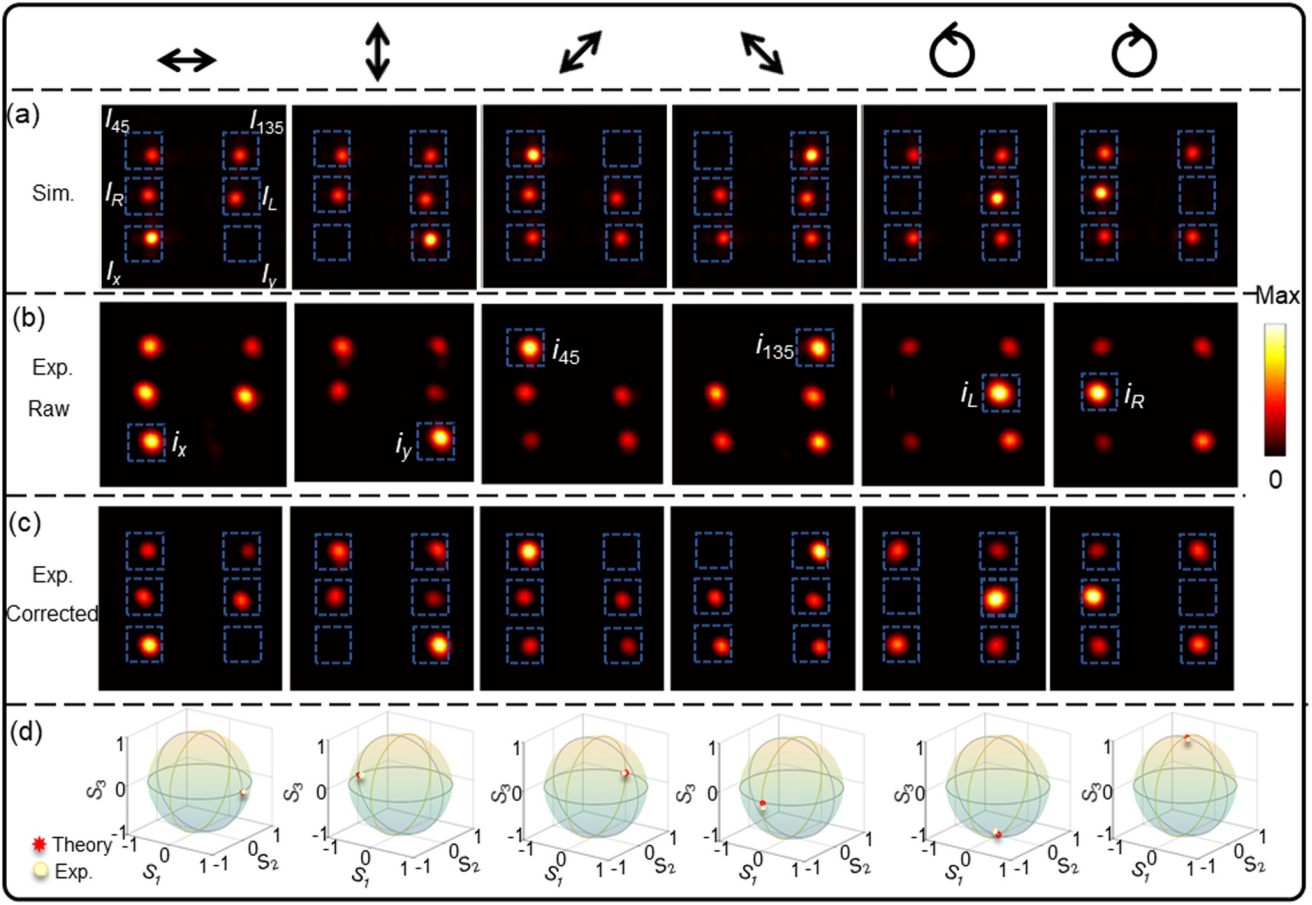
(a) Simulated light intensity distributions on the focal plane when the incident polarization is *x*‐, *y*‐, 45°‐, 135°‐, *L*‐, *R*‐. (b) Measured and (c) corrected light intensity distributions on the focal plane. (d) The normalized reconstructed (red stars) and theoretical (yellow circles) Stokes parameters on a Poincaré sphere at different polarization states.

For an arbitrary polarization, the full polarimetry process is displayed in Figure 3a. Initially, the intensities of the six focal spots can be obtained through a single‐shot measurement of the field distribution on the focal plane. Subsequently, the Stokes parameters can be efficiently reconstructed via matrix operations, enabling the rapid and accurate determination of the polarization state of the incident light. Based on this principle, three randomly selected incident lights with different polarization states are used for simulation analysis. For example, when the intensity distribution of the incident source is nonuniform, as shown in Figure 3b, the simulated field distributions in the focal plane of the metalens under different polarization states are shown in Figure 3c. The theory and reconstructed Stokes parameters are plotted in Figure 3d. For the quantitative characterization of polarization detection accuracy, we define the error function as

(5) $$r=\frac{1}{3}\sqrt{{\left(s_{1}-S_{1}\right)}^{2}+{\left(s_{2}-S_{2}\right)}^{2}+{\left(s_{3}-S_{3}\right)}^{2}}/s_{0}\times 100\%,$$

where *s* is the experimental result, and *S* is the theoretical value. The error functions of Figure 3c are 2%, 2.4%, and 2.3%, respectively. The reconstructed results are consistent with the theoretical values.

**FIGURE 3:**
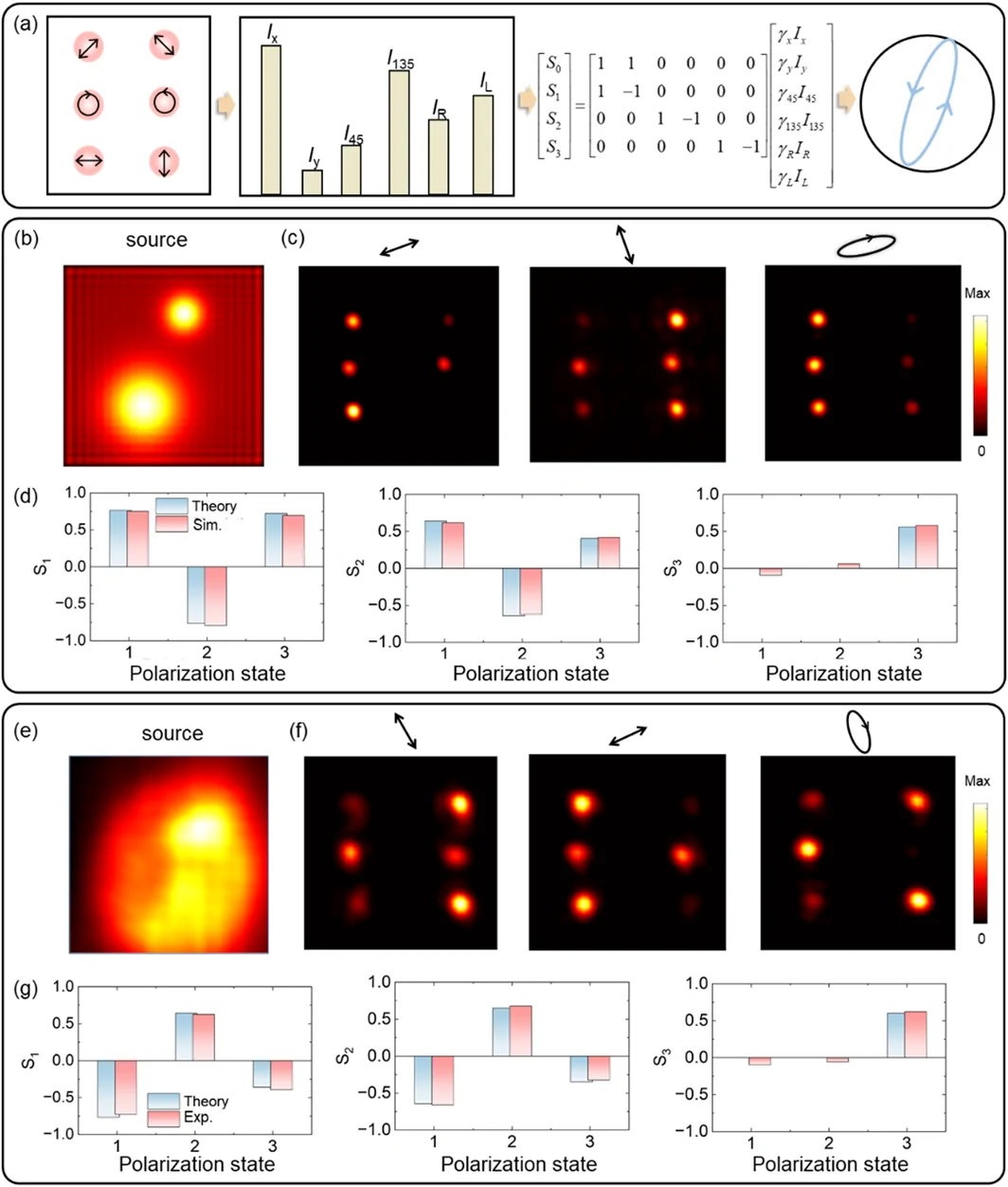
(a) Schematic diagram of the polarimetry process. (b–d) The simulated results for polarization analysis: (b) The intensity distributions of the source. (c) The intensity distributions on the focal plane for three random polarization states. (d) Theory and reconstructed Stokes parameters for three random polarization states. (e–g) The experiment results for polarization analysis: (e) The intensity distribution of the source. (f) The intensity distributions on the focal plane for three random polarization states. (g) Theory and reconstructed Stokes parameters for three random polarization states.

Subsequently, we experimentally characterize the performance of the designed polarimetry. By rotating a half‐wave plate or a quarter‐wave plate, the three distinct polarization states of incident light are randomly generated. The incident field distribution is shown in Figure 3e. The beam center is right‐shifted, and the beam is not a perfect Gaussian beam. The measured intensity distribution on the focal plane is shown in Figure 3f. The theoretical and reconstructed Stokes parameters are plotted in Figure 3g. The reconstructed results are consistent with the theoretical values. The error function of Figure 3g is 2.1%, 2.2%, and 2.2%, respectively. These results demonstrate the superior capability of metalenses in polarization detection under nonuniform intensity distributions. This approach exhibits significantly enhanced robustness compared to tessellated metalens designs under the same illumination conditions. Details are discussed in the Supporting Information S1: S1.

To validate the spatial polarization detection capability of the metasurface, we have redesigned and fabricated a 4 × 4 metalens array as a spatial polarization analyzer, as shown in Figure 4a. The whole design has the dimensions of 10 × 10 cm^2^ and a focal length of 3 cm. The spatial resolution, determined by the dimension of a single metalens, is 2.5 cm in the present design. Our simulations demonstrate that it can be minimized to 1.6 cm, below which the six focal spots on the focal plane can no longer be well resolved (Supporting Information S1: S2). To generate a specific polarization distribution over this aperture, a linear polarizer of the same size with 4 × 4 pixels is fabricated using laser direct writing technology, as shown in Figure 4b. Each pixel is a polarizer with metallic grating structures. The width of the grating and gap are both 40 μm. The transmission axis direction for each pixel is indicated by the yellow arrows in Figure 4d. In the experiment, a lens with a long focal length was employed to collimate the incident light into Gaussian light with enough beam waist to cover the entire surface. The *y*‐polarized incident light was converted into circularly polarized light through a QWP. Then, a spatially varying linear polarization state was generated using the linear polarizer in Figure 4b. This structured light was then incident upon the metasurface. The intensity distribution on the focal plane was measured by scanning point by point with a pyroelectric detector, and the measured results are shown in Figure 4c. Each sub‐lens generates six foci corresponding to six fundamental polarization components. The energy distribution of the focus varies from one pixel to another. It is worth noting that the signal‐to‐noise ratio in Figure 4c is significantly reduced compared to the uniform polarization detection. The reason is that the beam expansion leads to a decrease in the energy density rather than the performance of the metasurface itself. The correction coefficients for all sub‐lenses are [0.97, 1, 0.91, 0.9, 1.3, 1.3]. We further calculate the full Stokes parameters of each pixel. As shown in Figure 4e, the reconstructed results are highly consistent with the incident light. By calculating the polarization angle and ellipticity angle, the detected spatial polarization distributions are more intuitively depicted by the red dashed line in Figure 4d, which are almost consistent with the theoretical results. We define the total spatial error function as

(6) $$r_{\text{tot}}=\frac{1}{N}\sum_{i=1}^{N}r_{i},$$

where *N* is the number of pixels. The total error function *r*_tot_ of Figure 4d is 7%. To verify the stability of the spatial polarization analyzer, we rotated the linear polarizer counterclockwise by 90° and performed spatial polarization detection again. The detection results are shown in Figure 4f–h, and the spatial detection *r*_tot_ is 6.2%. It proves the reliable and stable spatial polarization analysis capability of the device.

**FIGURE 4:**
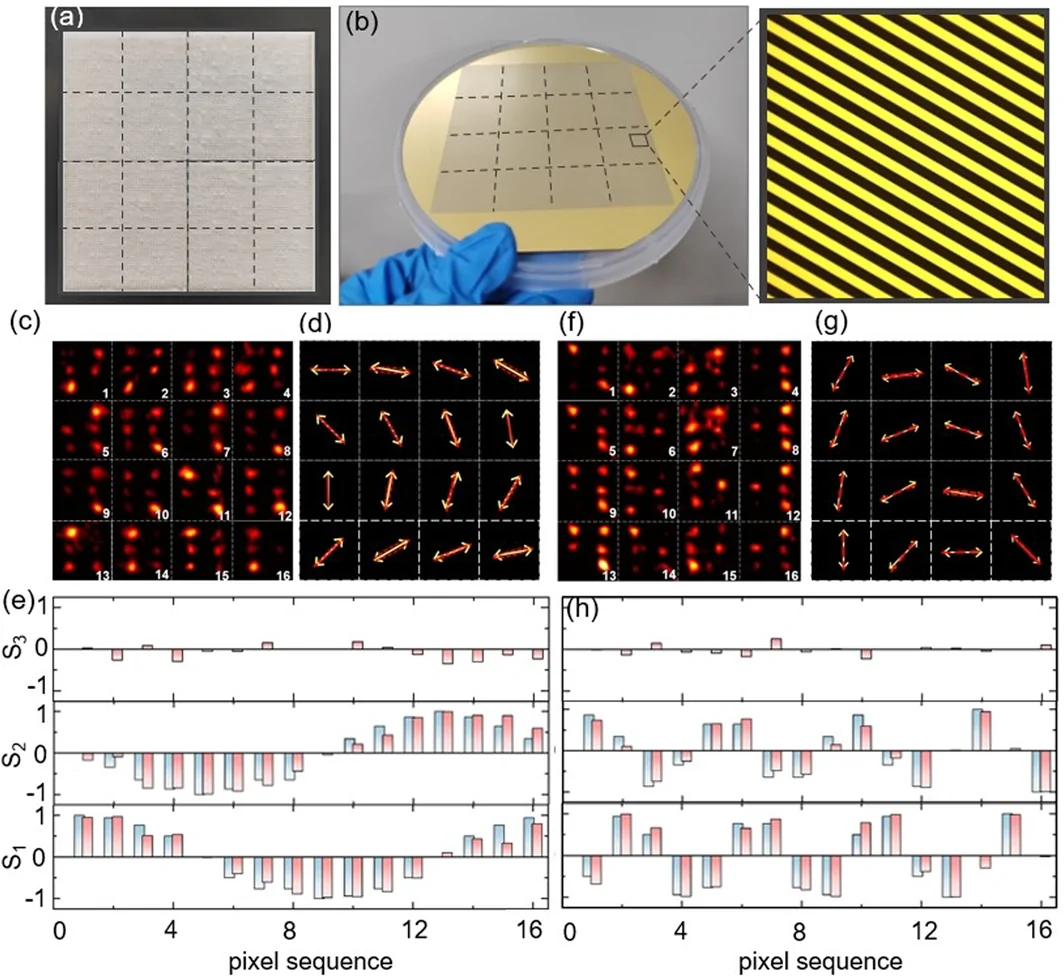
(a) The photograph of the 4 × 4 pixels polarization analyzer. (b) The photograph of the spatial linear polarizer and its details. (c) Intensity distribution on the focal plane. (d) The spatial polarization distributions. Yellow lines indicate reconstructed results, and red lines indicate the incident polarization states. (e) Theory and reconstructed Stokes parameters. (f–h) The polarization detection results when the linear polarizer is rotated 90° counterclockwise.

According to the above discussion, each metalens can generate 6 focal spots. A 2 × 2 array will generate a 6 × 4 spot array. The 6 focal spots from one metalens can be separated and interleaved with the spots from another metalens. For example, one type of focal spot distribution is shown in Figure 5a. Different colors represent the foci from different metalenses. The responsive polarization state is marked next to each focus. When the incident light has one of the six fundamental polarization states, some spots with orthogonal polarization will disappear, and the rest of the spots will be bright. This phenomenon enables the generation of six independent polarization‐sensitive image displays. As illustrated in Figure 5b, the field distributions under different polarization states reveal six distinct images corresponding to the characters “0”, “2”, “5”, “I”, “H”, and “+”. Furthermore, the proposed metalens holds significant potential for polarization imaging, as detailed in Supporting Information S1: S3.

**FIGURE 5:**
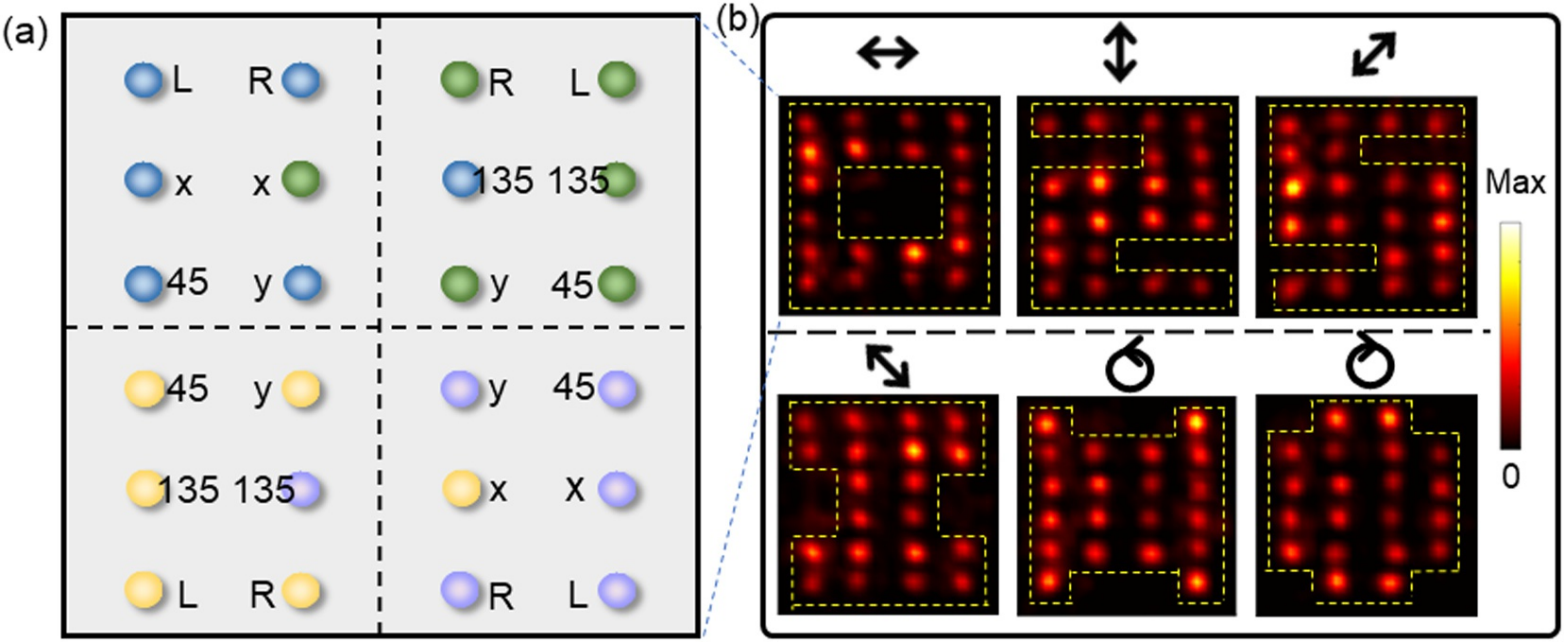
(a) Distribution diagram of metasurface focus. (b) The measured intensity distributions in different polarization channels.

## 4 Conclusion

In summary, we propose a new polarimetry method based on all‐dielectric compact metasurfaces. Four polarization‐sensitive meta‐atoms are integrated into a meta‐cell to split the incident light into six different polarization components and generate six off‐axis foci with good tolerance to nonuniform intensity distribution. By observing the intensity of each component, the Stokes parameters can be accurately obtained. In addition, the metasurface can be divided into multiple sub‐lenses to achieve spatial polarization distribution detection. We use a 4 × 4‐pixel metasurface for experimental verification, which confirms the feasibility of our proposed compact spatial polarimetry scheme. Finally, by controlling the spatial distribution of the foci and utilizing the extinction principle of orthogonal polarization channels, the designed metasurface achieves six dependent polarization‐sensitive image displays.

## 5 The Method of Digital Light Processing 3D Printing

The designed metasurface is fabricated by Digital Light Processing 3D printing. Firstly, photosensitive resins HDDA, HEA, and TMPTA, mixed in a 2:1:1 ratio, were combined with the photoinitiator TPO to prepare an organic solvent. Ceramic powder was then dissolved in this solvent to form the ceramic slurry. The slurry was injected into the printer tray. Under exposure to 405 nm UV light, the ceramic slurry was solidified layer by layer (20 μm thick, 10 s exposure per layer at 7 mW/cm^2^ power density) until printing was done. Finally, the ceramic embryo was heated at 600°C for 30 h in a degreasing furnace and at 1600°C for 20 h in a sintering furnace to obtain the ceramic sample.

## Author Contributions

**Shengnan Guan:** conceptualization, methodology, investigation, writing – original draft, data curation, visualization, software. **Jierong Cheng:** funding acquisition, writing – review and editing, validation, supervision, project administration, formal analysis, conceptualization. **Haifeng Xu:** software. **Fan Li:** software. **Chenglong Liu:** software. **Shengjiang Chang:** funding acquisition, writing – review and editing, supervision, resources.

## Supporting information


Supporting Information S1

## Data Availability

Data underlying the results presented in this work are not publicly available at this time but may be obtained from the authors upon reasonable request.
