# Supplementary material for: Terahertz Metasurface for Spatial Detection of Full Stokes Parameters and Polarization Imaging
Source: Nanophotonics. 2026 Apr 27;15(9):e70109. doi: 10.1002/nap2.70109 (PMC13642052; doi:10.1002/nap2.70109)
Supplement: Supplementary file 1 [file NAP2-15-e70109-s001.docx]

Supporting Information

Terahertz Metasurface for Spatial Detection of Full Stokes Parameters and Polarization Imaging

Shengnan Guan,^1^ Jierong Cheng,^1,2,3,^* Haifeng Xu,^1^ Fan Li,^1^ Chenglong Liu,^1^ Shengjiang Chang ^1,2,3^

**S1 Comparison analysis of the proposed subwavelength-integrated metalens and traditional tessellated metalens**

**Figure S1** (a) Schematic of the rectangular-tessellated metalens1. (b) Schematic of the subwavelength-integrated metalens2. (c) Simulated intensity distributions on the focal plane when the incident polarization is *x*-, *y*-, *45*°-, *135*°-, *L*-, *R*- for metalens1. (d) Simulated intensity distributions on the focal plane when the incident polarization is *x*-, *y*-, *45*°-, *135*°-, *L*-, *R*- for metalens2. (e) The non-uniform source. (f) Simulated focal spots of metalens1 with non-uniform illumination. (g) Simulated focal spots of metalens2 with non-uniform illumination. (h) Reconstructed Stokes parameters for two metalenses.

We compare the performance of the proposed subwavelength-integrated metalens and rectangular-tessellated metalens in terms of the same non-uniform illumination. For consistency, the sizes of the two metalenses are both 5 cm. The focal lengths are both 5 cm. The configuration of the two metalenses is illustrated in Figure S1(a) and S1(b).

We first find the correction coefficient for each metalens by uniform illumination of six known polarization states (*x-*, *y-*, *45°-, 135°-,* *L-*, *R-*). The focal spot distribution is shown in Figure S1(c) and S1(d). The correction coefficients are [1.1, 1, 0.87, 0.88, 0.79, 0.83] for the tessellated metalens1 and [1.62, 1, 1.05, 1.32, 1.1, 1.02] for the subwavelength-integrated metalens2.

Next, a source with a non-uniform intensity distribution is applied to both metalenses, as shown in Figure S1(e). The simulated field distributions in the focal plane of both lenses in Figure S1(f-g) are used for polarization characterization. The theoretical and reconstructed Stokes parameters are shown in Figure S1(h). The error is calculated to be 2% and 12%, respectively, for our metalens and the tessellated one, using the error function Equation 5 of the main text. The results clearly demonstrate the enhanced robustness of the proposed subwavelength-integrated metalens.

**S2 Polarization imaging resolution and array size**

**Figure S2** The focusing efficiency and intensity distribution in the focal plane when the metalens has different sizes.

We design a series of metalenses with different diameters (*L*). Under *x*-polarized incidence, the simulated focal plane field distribution and focusing efficiency are shown in Figure S2. The six focal spots are hardly distinguishable when *L*=1 cm. When *L*=1.6 cm, the metalens maintains good performance with a focusing efficiency above 75%, and the six focal spots are clearly separated without obvious crosstalk. Therefore, the minimum spatial resolution is approximately 1.6 cm. In this case, each sub-metalens only contains 8×8 meta-units.

Theoretically and from the perspective of fabrication technology, there is no fundamental limitation on scaling the metalens array to larger sizes or higher pixel densities. However, in practical applications, several key challenges need to be addressed. First, the THz source power is inherently limited. Scaling the array to a larger size requires a larger incident beam size, which inevitably leads to a decrease in power density across the array. Second, the detector’s responsivity is finite. If the power density is too low, the focal spot intensity will be weakened, resulting in a reduced signal-to-noise ratio (SNR). This degraded SNR will introduce errors into polarization state analysis and compromise the accuracy of the reconstructed Stokes parameters.

**S3 Numerical study of polarization imaging.**

**Figure S3** The schematic diagram of the polarization imaging system.

To demonstrate the feasibility of the proposed metalens for polarization imaging, we performed numerical simulations. Figure S3 illustrates the polarization imaging system, which is a classical 4*f* system. The distances between the object, lens1, lens2, and imaging plane are *f*, 2*f*, and *f*, respectively. The objects are letters “I” and “E” with spatially dependent polarization response. When the plane wave passes through the object, the wave with a certain spatial polarization distribution is collected by the lens1 and separated onto the focal plane. Then the focal spots further illuminate the lens2 and are imaged on the 4*f* plane. The lens1 is the proposed subwavelength-integrated metalenses, and the diameter is 10 cm. The lens2 is a traditional plano-convex lens, and the diameter is also 10 cm. The calculated imaging results in the imaging plane are shown in Figure S4(b), and the full-Stokes images are reconstructed in Figure S4(c). It confirms the ability of polarization imaging.

**Figure S4** (a) The raw imaging objects with spatial-dependent polarization features. (b) The imaging plane distribution. (c)The full-Stokes images.
